# Clinical Application of Next-Generation Sequencing for Molecular Classification in the Management of Endometrial Cancer: An Observational Cohort Study

**DOI:** 10.3390/cancers17111806

**Published:** 2025-05-28

**Authors:** Sabrina Paratore, Angela Russo, Giusi Blanco, Katia Lanzafame, Eliana Giurato, Giovanni Bartoloni, Marco D’Asta, Mirella Sapienza, Valeria Solarino, Valentina Vinci, Giulia Maria Bonanno, Giuseppe Ettore, Roberto Bordonaro

**Affiliations:** 1 Department of Pathological Anatomy, ARNAS Garibaldi Hospital, 95122 Catania, Italy; angelarct@tiscali.it (A.R.); egiurato@arnasgaribaldi.it (E.G.); gbartoloni@arnasgaribaldi.it (G.B.); 2 Department of Medical Oncology, ARNAS Garibaldi Hospital, 95122 Catania, Italy; gblanco@arnasgaribaldi.it (G.B.); klanzafame@arnasgaribaldi.it (K.L.); rbordonaro@arnasgaribaldi.it (R.B.); 3 Department of Obstetrics and Gynecology, ARNAS Garibaldi Hospital, 95122 Catania, Italy; mdasta@arnasgaribaldi.it (M.D.); msapienza@arnasgaribaldi.it (M.S.); giuliabonanno@arnasgaribaldi.it (G.M.B.); gettore@arnasgaribaldi.it (G.E.); 4 Radiotherapy Unit, ARNAS Garibaldi Hospital, 95122 Catania, Italy; vsolarino@arnasgaribaldi.it; 5 Radiology Unit, ARNAS Garibaldi Hospital, 95122 Catania, Italy; vvinci@arnasgaribaldi.it

**Keywords:** endometrial cancer, molecular profile, clinicopathological features, POLE mutated, mismatch repair-deficient, TP53mutated, no specific molecular profile

## Abstract

Background/Objectives: Endometrial cancer (EC) is the most common malignancy of the female genital tract. In 2013, The Cancer Genome Atlas analyzed the molecular profile of endometrial tumors identifying four risk classes (POLE ultramutated, mismatch repair-deficient, copy-number low-microsatellite stable, and copy-number high-serous-like. This classification is reshaping the current understanding of EC, enabling more refined risk stratification and uncovering potential therapeutic targets tailored to specific molecular subgroups. In the context of these four categories, it is possible to identify different molecular alterations that correlate with different prognoses. Methods and Results: We retrospectively analyzed tissue samples from eighty-five EC patients, performing multigene profiling using a 50-gene next-generation sequencing (NGS) panel to categorize them into distinct molecular subtypes; we observed the following distribution: 5.9% POLE, 25.8% mismatch repair-deficient/microsatellite instability (MMRd/MSI), 11.8% p53abn/TP53mut, and 56.5% NSMP. A favorable concordance (97.6%) was shown in MSI NGS-based analysis and MMR IHC results, and the agreement rate of p53 IHC and *TP53* mutation was 92.3%. When we analyzed the correlation between molecular subtypes and clinicopathological features, we found that molecular subtypes significantly differentiated by grade, FIGO stage, and lymphovascular invasion (LVSI). These findings seem to support the effectiveness of our NGS-based classifier and its reliability in distinguishing both MSI and TP53 mutated cancers. This study also explored mutations in *PIK3CA*, *PTEN*, *KRAS*, *ERBB2*, and *ESR1* genes, noting their potential as targets for treatments. *PIK3CA* mutations were linked to favorable features, such as early disease stage and absence of LVSI. Conclusions: Our study highlights the potential of a medium-complexity NGS panel for supporting the molecular classification of endometrial cancer, complementing the existing diagnostic algorithms. By identifying additional biomarkers, we provided valuable insights into the genomic landscape of EC. However, further exploration of the molecular profiles is needed to validate these findings and improve the identification of patients at a higher risk of unfavorable outcomes.

## Simple Summary

Endometrial cancer (EC) is the most frequent gynecologic malignancy, and recent molecular classifications have improved our understanding of its behavior and prognosis. In this study, we analyzed tumor samples from 85 EC patients using a targeted 50-gene next-generation sequencing (NGS) panel to classify tumors into four molecular subtypes: POLE mutated, mismatch repair-deficient (MMRd), p53-abnormal, and no specific molecular profile. Our results show a strong agreement between NGS and standard immunohistochemical methods for identifying MMRd and p53-abnormal cases. Molecular subtypes were significantly associated with clinical features, such as tumor grade, FIGO stage, and lymphovascular invasion. Additionally, we identified frequent mutations in genes like *PIK3CA*, *PTEN*, and *KRAS*, some of which may represent potential therapeutic targets. This study supports the utility of a medium-complexity NGS panel in EC classification and guiding personalized treatment strategies.

## 1. Introduction

Endometrial cancer (EC) is the most common malignancy of the female genital tract in Europe. There were 417,397 new cases of EC reported worldwide in 2020. The incidence in Italy is 14.4 per 100,000 and mortality is 2.2 per 100,000 [1]. In the past, treatment decisions and prognosis were based on the histopathology of EC, including lymphatic vascular space invasion (LVSI), histological type, grade, and stage of disease.

However, in 2013, an integrated molecular characterization of EC by The Cancer Genome Atlas (TCGA) Research Network showed that EC could be subdivided into four subtypes with different prognostic implications on the basis of mutational burden and somatic copy-number variations [2]. Subsequent studies have identified immunohistochemical (IHC) and molecular tests as surrogates for genomic analyses performed by TCGA [3,4,5,6,7,8]. These advancements have led to the identification of four distinct EC molecular prognostic subgroups: POLE ultramutated (POLEmut), mismatch repair-deficient/microsatellite instability (MMRd/MSI), p53 abnormal (p53abn), and no specific molecular profile (NSMP). This molecular classification system, based on TCGA’s findings [2], was further refined through the Proactive Molecular risk Classifier for Endometrial Cancer (ProMisE) trial, making it an essential tool for clinical practice [6,7,8]. In a significant advancement for EC management, the International Federation of Gynecology and Obstetrics (FIGO) incorporated molecular information into its 2023 FIGO staging system, defining specific substages by molecular signature to stratify the risk and identify the therapeutic strategy [9,10,11]. The ProMisE/TransPORTEC (Post-Operative Radiation Therapy in Endometrial Carcinoma) molecular classification systems define the prognosis of ECs, but not all molecular signatures of these tumors fall into well-defined subgroups in terms of prognosis and therapeutics. The majority of the EC population belongs to the NSMP subtype (39–60%), with an overall intermediate clinical behavior, for which prognosis and the management of adjuvant interventions still rely on the evaluation of traditional clinicopathologic features. It is still a challenge to identify molecular markers that may guide therapeutic decisions in this setting of patients. Additionally, the current molecular classification systems, such as ProMisE/TransPORTEC, depend on multiple testing platforms, including comprehensive genomic profiling, IHC testing, and polymerase chain reaction (PCR) assays. These multiple methods, while highly informative, can be expensive, time-consuming, and labor-intensive. In clinical practice, these hurdles may hinder the routine adoption of these molecular classifications, particularly in settings characterized by limited resources.

In light of these challenges, next-generation sequencing (NGS) technologies have emerged as a promising tool for molecular subtyping in EC, and for further exploration. Several studies have already demonstrated the potential of NGS in EC molecular classification, offering a more accessible and affordable solution for routine clinical use [9,10]. By analyzing a broad range of genetic markers at once, NGS provides more comprehensive and accurate insights compared to traditional methods, like IHC or polymerase chain reaction (PCR). Furthermore, the current development of simplified NGS panels would allow for a more efficient and cost-effective way to diagnose and classify EC subtypes, reducing the reliance on multiple, complex tests.

In this study, we develop a pragmatic NGS-based classification model to effectively stratify a cohort of EC patients into distinct molecular subgroups. By correlating molecular data with clinicopathological features, we aim to gain deeper insights into the prognostic and predictive roles of the genomic landscape of EC. Additionally, this approach has the potential to uncover potential molecular targets, particularly for the NSMP subgroup, which currently lacks effective targeted treatment options.

## 2. Materials and Methods

### 2.1. Case Selections

Our retrospective cohort consisted of 85 patients with ECs treated at the ARNAS Garibaldi Nesima Hospital from January 2023 to June 2024. According to the revised standards of the 5th edition of the WHO classification in 2017, all hematoxylin and eosin slides were reviewed by two pathologists and the diagnosis of EC was confirmed on the basis of morphologic features. Clinical and pathology databases of patients were collected, including age, histological subtypes, grade, FIGO stage, LVSI, and lymph node status. Informed consent to the data collection and analysis was obtained from all patients according to the institutional guidelines.

### 2.2. Next-Generation Sequencing (NGS) Assay

To identify molecular subgroups and investigate the genomic profile of EC patients, all samples (*n* = 85) were analyzed by NGS assay. In particular, six formaldehyde-fixed and paraffin-embedded tissue (FFPE) sections of 5- to 10 μm thin sizes containing more than 50% tumor cells were used, and total DNA was extracted from FFPE using a EZ1 e EZ2 DNA FFPE Kit following the manufacturer’s instructions (Qiagen, Germany). The DNA concentration was measured using a Qubit Fluorometer (ThermoFisher Scientific, Waltham, MA, USA 02451) and DNA degree of fragmentation was carried out using EasyPGX qPCR and EasyPGX Analysis Software version 4.0.15 (Diatech Pharmacogenetics, 60035 Jesi (AN), Italy). All samples showed good-quality parameters and suitable for sequencing. For NGS analysis, the initial amount of DNA required to construct the library was between 10 and 50 ng. Library preparation with hybridization capture-based target enrichment was conducted using a commercially available 50-gene panel Myriapod NGS Cancer probe plus (Diatech Pharmacogenetics, 60035 Jesi (AN), Italy), following the manufacturer’s protocol. This panel covered hotspot mutation regions relevant to EC (including the TP53 and POLEexonuclease domains) and allowed for MSI analysis and for the evaluation of copy number variations (CNVs) (Supplementary Data, Table S1). Separate sequencing runs were performed by MiseqDx platform (Illumina Centre, Cambridge CB21 6DF, UK) and analyzed by Myriapod NGS Data Analysis Software v 5.0.10. Sequencing metrics along with alignment quality are summarized in Supplementary Data, Table S2; no technical replicates were performed. Data analysis focused on non-synonymous variants having functional consequences at the protein level (synonymous variants and intronic variants not affecting splice sites were excluded from the final analysis). Variants were further filtered based on the following criteria: a minimum read depth of 200X; variant allele frequency (VAF) threshold of at least 5%; presence in both forward and reverse reads; exclusion of known sequencing artifacts and common polymorphisms (using databases such as gnomAD); annotation using curated databases (e.g., COSMIC, ClinVar); and bioinformatic pipeline (Varsome clinical v.12.9.0 software) to assess pathogenicity (deleterious variants and uncertain significance variants (VUSs)). Regarding MSI analysis, MSI phenotype detection was based on the read-count distribution of 120 specific microsatellite loci. A given threshold was set using the coverage ratio of a specific set of repeat lengths for each microsatellite locus. The locus was categorized as unstable (MSS) if the coverage ratio was less than the given threshold. The MSI status was based on the percentage of unstable loci in the specific sample. A tumor sample was considered highly unstable (MSI-H) if >30% of the marker loci were length-unstable. Furthermore, gene-level copy number estimates were obtained by comparing the normalized read depth of each target region to a reference baseline generated from a pool of normal samples. Log2 ratio thresholds were applied to define amplifications and deletions (gains log2 ratios > 1 and losses at <−1, CNVs score).

### 2.3. Immunohistochemistry

IHC was performed on 3 μm-thick FFPE sections of 85 EC samples using primary antibodies as follows: MLH1 (clone: M1, Ventana Medical Systems, Arizona, USA), PMS2 (clone: A16-4, Ventana Medical Systems, Arizona, USA), MSH2 (clone: G219-1129, Ventana Medical Systems, Arizona, USA), and MSH6 (clone: SP93, Ventana Medical Systems, Arizona, USA) ready to use on BenchMark IHC/ISH instruments with the OptiView DAB IHC Detection Kit, OptiView Amplification Kit, and ancillary reagents. MSI status was identified by the EASY PGX READY MSI KIT in the Real-Time PCR EASY PGX method (Diatech Pharmacogenetics, Italy), including 8 mononucleotide repeat MSI markers (BAT25, BAT26, NR-21, NR22, NR-24, NR27, CAT25, and MONO-27) according to these criteria: MS-stable (MSS, no, or one MSI marker) and MSI-H (MSI-H, two, or more MSI markers) tumors without a matched normal sample [12]. MSI analysis was also confirmed by the NGS DNA panel.

Abnormal/aberrant/mutation-type p53 (p53abn/TP53mut) was identified by IHC and NGS-based analysis. IHC was performed using primary-antibody anti-p53 (DO-7, Ventana Medical Systems, Tucson, Arizona 85755, USA). Following the previous published criteria [13], three p53 expression patterns were considered aberrant: strong diffuse nuclear staining in >80% of tumor cells; complete absence of staining; and cytoplasmic staining.

Immunostaining for HER2 with the anti-HER2/neu clone 4B5 (Ventana, Medical Systems, Tucson, Arizona 8575, USA) was also performed in four patients with advanced-stage EC at diagnosis.

### 2.4. Statistical Analysis

Statistical analysis was performed by SPSS 22.0 software. At the univariate level, correlations of clinicopathologic features (age, histological type, FIGO stage, grade, LVSI, and lymph node status) with molecular subtypes and *PIK3CA* mutations were tested using χ^2^ statistics or the Fisher exact test in the case of categorical and *t*-tests or ANOVA for continuous variables. A multivariate analysis was conducted using a general linear regression model, including only variables that were statistically significant in the univariate analysis (*p* ≤ 0.05). A *p*-value of ≤0.05 was considered statistically significant. The collection and visual analysis of alteration data were implemented in Heatmap using Oncoprinter by cBioPortal for Cancer genomics (https://www.cbioportal.org/ accessed on 15 February 2025 1:15:28 PM).

## 3. Results

### 3.1. Clinicopathological Characteristics

A total of 85 patients diagnosed with EC with a median age at diagnosis of 64 years (38–90 years old) were enrolled in the present study. All patients underwent total hysterectomy with bilateral salpingo-oophorectomy and lymph node staging (sentinel lymph node biopsy vs. lymphadenectomy). The histological types were distributed as follows: 84.7% endometrioid, 9.4% serous histological type, 4.7% carcinosarcoma, and 1.2% clear cell.

According to the 2009 International FIGO staging criteria, there were 26 (30.6%) patients in stage IA, 36 (42.3%) in stage IB/II, 15 (17.7%) patients in stage III, and 8 (9.4%) in stage IV. The baseline clinicopathological characteristics of EC patients are shown in Table 1. Patients were molecularly classified based on genomic features in the following subtypes: POLEmut group, MMRd/MSI group, and p53abnormal/TP53mutated (p53abn/TP53mut) group. In the NSMP, all molecular signatures that did not fall into the previous groups were considered.

In terms of postoperative adjuvant therapy (Table 1), 12 (13.9%) patients were treated with chemo-radiotherapy and vaginal brachytherapy; 19 (22%) with external beam radiation therapy and brachytherapy; 2 (2.3%) with vaginal brachytherapy only; 1 (1.1%) patient refused any treatment; and 1 (1.1%) refused external radiotherapy. No patient relapsed or died from EC. The correlation of molecular data to clinical outcomes was not performed because EC patients were predominantly in early disease stages and had a short follow-up period (median of 18 months).

### 3.2. Molecular Subtyping

**Figure 1:**
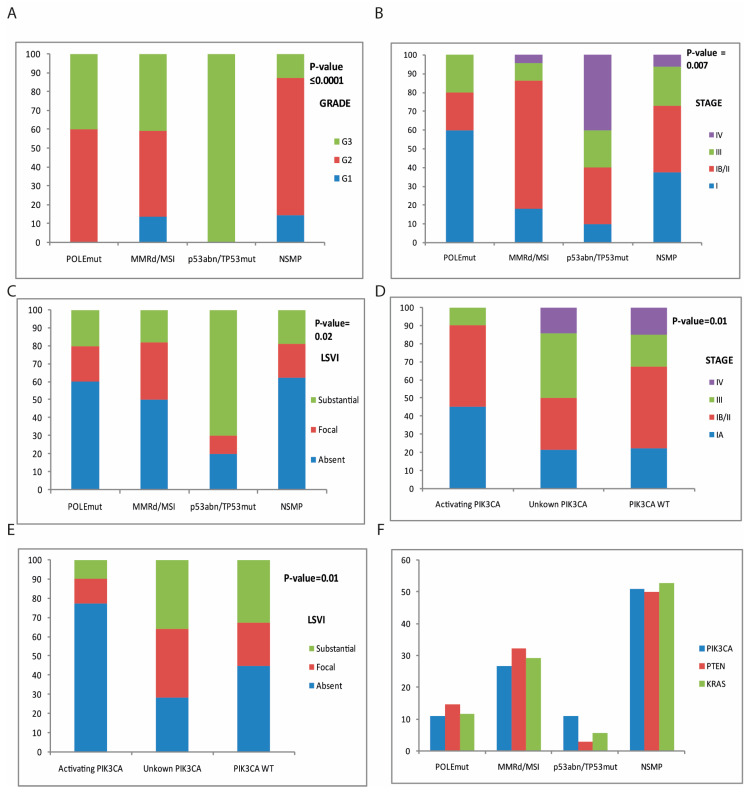
Clinicopathologic and molecular features of EC patients. Bar plot comparing (**A**) grade, (**B**) FIGO stage, and (**C**) LVSI among the molecular subtypes. Bar plot comparing (**D**) FIGO stage and (**E**) LVSI among ECs cases with active *PIK3CA* mutations, unknown *PIK3CA* mutations, and *PIK3CA* WT. (**F**) Distribution of *PIK3CA*, *PTEN*, and *KRAS* mutations among EC molecular subtypes.

Utilizing TCGA/ProMisE molecular classification criteria, we categorized patients into four molecular subgroups: POLE (5.9%), MMRd/MSI (25.8%), p53abn/TP53mut (11.8%), and NSMP (56.5%) (Table 1, Figure 1). We then explored the relationship between molecular classification and clinicopathological features (age, histological type, FIGO stage, grade, LVSI, and lymph node status) by univariate analysis, as shown in Table 1. There were no statistically significant variations between subgroups in terms of age distribution and lymph node status. However, significant differences were found among subtypes by histological type, grade, FIGO stage, and LVSI. When we performed multivariate analysis using a general linear regression analysis, the overall model was statistically significant for all four dependent variables: histological type (F (4, 79) = 5.692, *p* = 0.01, Adjusted R^2^ = 0.224), grade (F (4, 79) = 7.942, *p* = 0.001, Adjusted R^2^ = 0.287), FIGO stage (F (4, 79) = 2.555, *p* = 0.035, Adjusted R^2^ = 0.115), and LSVI (F (4, 79) = 3.610, *p* = 0.05, Adjusted R^2^ = 0.155) (Table 1).

### 3.3. POLE

According to the criteria described in previous studies [14,15], the POLEmut group was defined by mutations in the exonuclease domain, which were identified through NGS analysis. Of the five POLEmut patients, four POLE tumors harbored the most common hotspot missense mutations, P286R, V411L, S297F, A456P; the remaining one case had the less-frequent variant D275G. POLE mutant-group patients had a median age 56 years. They were associated with endometrioid histological type, a tendency toward a higher grade (G2 and G3, Table 1) but lower stage, and LSVI absence than other groups (Figure 1A–C), however without reaching statistical significance (POLEmut vs. MRRd/MSI; vsp53abn/TP53mut; vs. NSMP *p*-value > 0.05). Of note, two cases in the POLE group were multiple-classifiers; in addition to harboring *POLE* common alterations, they exhibited MMRd/MSI and showed focal and substantial LSVIs.

### 3.4. MMRd/MSI

The MSI group was characterized by the presence of microsatellite instability evaluated by Real-Time PCR and NGS analysis and/or by the identification of MMRd evaluated by IHC. Most MSI cases (91%, Table 1) had endometrioid histology, were early-stage I–II (86.4%, Table 1, Figure 1B), and showed a trend toward a high grade, significantly greater than the NSMP group (grade MSI vs. NSMP *p*-value = 0.02, Figure 1A), but lower than the p53abn/TP53mut group (grade MSI vs. p53abn/TP53mut *p*-value 0.001, Figure 1A). In addition, MSI cases mainly showed an absent or focal LSVI (Figure 1C). Furthermore, among MSI-group cases, three patients with high-grade serous, endometrioid, and carcinosarcoma histotypes were multiple-classifiers harboring the molecular feature MMRd/MSI-p53abn/TP53mut.

### 3.5. p53abn/TP53mut

The p53abn/TP53mut subtype was present in patients with a median age of 70 years, and poorly differentiated serous carcinomas histological types (Table 1). Most of the cases were at FIGO stages III–IV (60%) and showed a substantial LSVI (Table 1), which was significantly the highest among all groups (p53abn/TP53mut vs. MSI *p*-value 0.01; p53abn/TP53mut vs. NSMP *p*-value = 0.004, Figure 1C, Table 1).

### 3.6. NSMP

The NSMP group encompassed the largest and most heterogeneous group of EC cases. Among NSMP tumors, 44 were classified as endometrioid carcinomas (92%) and 4 were of other histologies (8%, Table 1), specifically 1 serous carcinoma, 1 clear-cell carcinoma, and 2 carcinosarcomas. In this subtype, the median age of diagnosis was 64 years. Most NSMP patients were early-stage I/II (73%, Table 1), grade G2 (73%), and lymph node-negative (87.5%). However, in this subgroup, we found the highest number of patients with lymphatic positivity compared to the other groups (12.5%, Table 1). Only nine patients showed LSVIs (18.7%, Table 1).

### 3.7. Concordance Between IHC, NGS, and PCR Results

**Figure 2:**
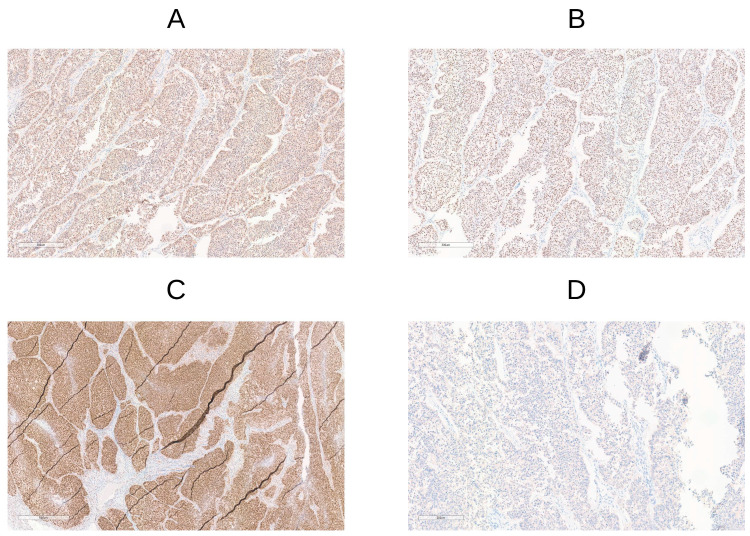
Discordant case in which the Real-Time PCR method indicated Microsatellite Stability (MSS), but the MMR protein IHC was abnormal. Tumoral cells showed a reduced expression of (**D**) PMS2 protein with normal nuclear staining for (**A**) MSH2, (**B**) MSH6, and (**C**) MLH1 proteins. This observation is in agreement with NGS analysis and is associated with Lynch Syndrome.

For the MSI/MMRd status assessment, we compared different molecular testing methods, including IHC, PCR-based, and NGS analyses. The majority of cases (97.6%) exhibited a high level of agreement between IHC and NGS results. A single discordant case was observed where the Real-Time PCR method indicated Microsatellite Stability (MSS), but IHC indicated a MMRd status with a loss of PMS2 expression (Figure 2). However, validation with NGS confirmed that IHC and NGS results were consistent, suggesting that the PCR-based results might have been an outlier or misinterpretation. Furthermore, germinal analysis of mismatch repair genes indicated Lynch Syndrome. Another case showed weak and focal MSH6 staining by IHC and MSS (with only the MONO27 locus unstable) by Real-Time PCR. However, NGS analysis demonstrated extended instability, suggesting that NGS might be a sensitive tool for detecting MSI status, especially for solving cases that are difficult to interpret.

Furthermore, we studied the concordance between the expression of p53 and *TP53* lteration by IHC and NGS methods.

**Figure 3:**
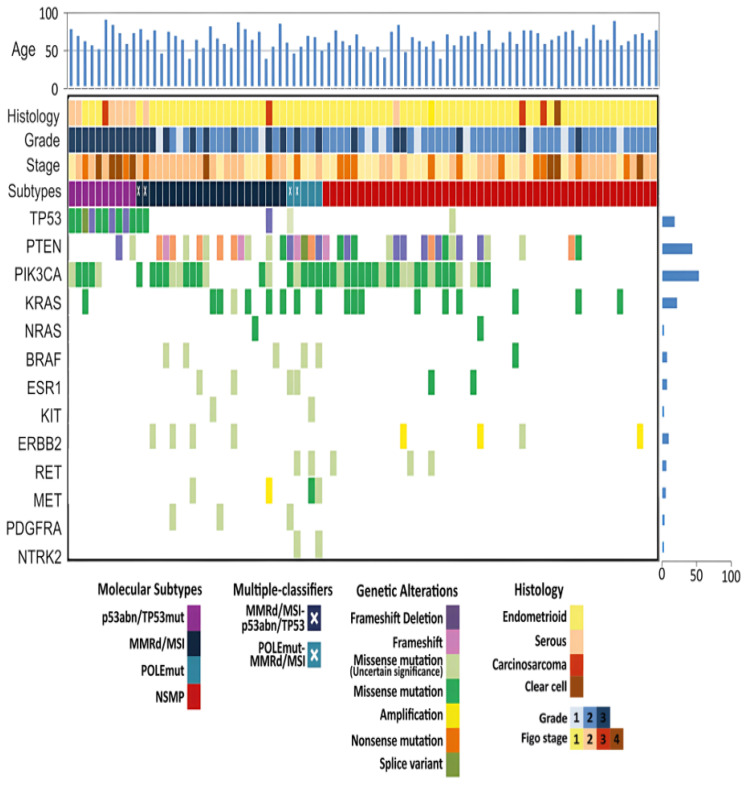
Genomic profiles of EC patients with different molecular subtypes. In the heatmap, each column represents a tumor and the rows show alterations for each gene. The bottom part of the graph shows the summary of the histopathologic and clinical information for each case. The bar graph at the top depicts the sample’s age. The bar graph on the right of the panel shows the number of alterations for each gene. Mutation types and clinicopathologic features are color-coded according to the legend.

Indeed, it has previously been reported that a variability in concordance rates (ranging from 60% to 92.3%) could be attributed to different definitions of p53abn and *TP53* alterations across various studies [16]. In our work, we considered *TP53* functional mutations for molecular classification (excluding synonymous and uncertain significance variants). Under these criteria, IHC and NGS provide largely consistent results with a concordance rate of 92.3% (Figure 3). All p53abn cases by immunohistochemistry harbored pathogenic *TP53* alterations by NGS. Interestingly, only a case with a *TP53* frameshift mutation (NM_000546.6:c.140delC, p.P47Rfs*76), showed wild-type staining patterns by IHC and was multi-classifier MMRd/MSI-p53abn/TP53mut. The exception noted in the multi-classifier case underscores the importance of using the NGS method for a more comprehensive assessment of *TP53* alterations.

### 3.8. Identification of Additional Biomarkers

Beyond the TCGA/ProMisE subtypes, we identified additional biomarkers with different roles. As shown in Figure 3, our cohort was characterized by genomic alterations in several genes, including *PIK3CA* (53%), *PTEN* (42%), *KRAS* (21%), *ERBB2* (9.4%), *ESR1* (7.1%), *BRAF* (7.1%), *RET* (5.9%), *PDGFRA* (3.4%), *NRAS* (2.4%), *MET* (4.7%), *KIT* (2.4%), and *NTRK2* (2.4%) genes. Note that more than half of EC patients harbor alterations in the *PIK3CA* gene. The mutations of *PIK3CA* localized in hotspots centered on p110-α functional domains. These included mutations in the N-terminal region—particularly the p85/adaptor-binding domain and the protein kinase C homology-2 (C2) domain (exons 1–7)—as well as alterations within the helical and kinase domains in the C-terminal region (exons 9–20) (Table 2). Consistent with the findings from other studies [17,18,19], the majority of *PIK3CA* alterations (31/45, 68.8%) were gain-of-function mutations that activated the PI3K/AKT1/MTOR signaling pathway (Supplementary Data, Table S3). Common activating mutations included R88Q, C420R, E545G, and H1047R; less frequent mutations, such as E545A, H1047Y, and M1043I (as reported in the TCGA and COSMIC databases) were also identified. The remaining mutations 14/45 (31%) have not been functionally characterized. Among these, Y1021C was recurrent in this study and in others, suggesting that it may confer a selective advantage in tumorigenesis, whereas A1035V was rare [20,21]. Additionally, functionally unknown mutations localized into the ABD and C2 domains, E81K, K111E, E110del, K111del, and V344A, were previously observed at low frequencies across various tumor types, with a particular relevance to ECs. The I102del mutation was specifically found in the TCGA database linked to EC. These data demonstrate that, in EC, the majority of somatic mutations occur in the amino-terminal domain of p110-α, unlike other types of tumors (colorectal, breast, or bladder carcinomas) [22].

To investigate the effects of *PIK3CA* mutations on clinicopathological features, we correlated cases with activating, unknown, and wild-type (WT) *PIK3CA* mutations with different parameters—age, histotype, grade, FIGO stage, lymph node status, and LVSI—by univariate analysis (Table 2). The age range for patients with *PIK3CA*-activating and unknown mutations was 38–86 years and 40–80 years, respectively (median: 61 and 62 years), while WT cases were in the range of 46–90 years (median: 67 years). No significant differences in age, histotype, grade, or lymph node status were observed between *PIK3CA* mutated and WT cases. However, nearly all *PIK3CA* mutated cases were in the early FIGO stage (Figure 1D, Table 2, *p*-value = 0.05). In addition, *PIK3CA*-activating mutations were predominantly associated with the absence of LVSI, while *PIK3CA*-unknown mutations and WT cases showed a significant tendency toward focal and substantial infiltration (Figure 1E, *p*-value 0.01). The association between *PIK3CA* mutations and LSVI were further confirmed—even if with a moderate effect size—by multivariate linear regression analysis (*p*-value = 0.05, Adjusted R^2^ = 0.096, Table 2). Furthermore, as previously reported, *PIK3CA* alterations overlapped with all POLE cases, with *PTEN* mutations in 78% (28 out of 36) and *KRAS* mutations in 39% (7 of 18) of cases, respectively [17,23,24]. *PTEN* mutations were mainly frameshift deletions that occurred across all coding regions; *KRAS* mutations were missense mutations (G12C, G12D, G12V, G13D, and G12S) confined in the tyrosine kinase domain (see Supplementary Data). The coexistence of both *PTEN* and *PIK3CA* mutations was statistically significant (*p*-value = 0.002, by *t*-test), suggesting potential synergistic effects on the over-activation of the PI3K downstream signaling pathway (Figure 1F). On the other hand, there was no significant difference in the frequency of *KRAS* mutations between *PIK3CA* activating, unknown mutant, and *PIK3CA* WT ECs (*p*-value = 0.93, by *t*-test), suggesting that *KRAS* and *PIK3CA* alterations may play different roles in endometrial carcinogenesis. Notably, in our cohort, we observed mutually exclusive *KRAS* and *TP53* alterations, with the exception of one case that harbored both alterations. Although *KRAS* and *TP53* mutations generally characterize separate molecular pathways, uncommon instances may involve both, indicating a merging of pathways in tumor development. In our 50-panel NGS analysis, intriguing findings also regarding *ERBB2* (HER2) and Estrogen Receptor 1 (*ESR1*) variants were obtained. Three NSMP patients exhibited an amplification of the *ERBB2* gene (defined by a CNV gain score ≥ 1 in more than 77 gene regions), which is a well-established target for anti-HER2 therapies in EC patients. Additionally, two NSMP patients presented hotspot mutations (Y537S, E380Q) in the ligand-binding domain of the *ESR1* gene, which encodes the estrogen receptor alpha (ERα). These *ESR1* alterations are linked to hormone therapy resistance, particularly in breast cancer [25,26]. In primary EC, *ESR1* alterations have previously been associated with poorer prognosis compared to wild-type *ESR1* tumors, emphasizing its potential role in the aggressiveness of the disease [25,26,27]. Additionally, *ESR1* mutations have been reported in EC patients treated with an aromatase inhibitor.

## 4. Discussion

Histology of EC is important in defining treatment strategies, especially in adjuvant therapy. However, pathological classification by histological subtype and grade has been criticized for its subjectivity and reproducibility challenges, which may complicate the clinical decision-making process [28]. Since 2013, the TCGA classification of EC has shifted its focus to integrating molecular signatures with histology. However, due to difficulties in applying TCGA to clinical settings, other classification systems, such as ProMisE and TransPORTEC, have been proposed. These systems combine IHC for p53 and the MMR proteins with *POLE* exonuclease-domain mutant-hotspot sequencing. ProMisE and TransPORTEC stratify risk into four groups, like TCGA [3,4,8,29]. According to these data, POLEmut EC patients can avoid the adverse effects of adjuvant therapy, whereas MMRd EC patients benefit from radiotherapy but not from chemotherapy. A retrospective analysis of the PORTEC3 study demonstrated a PFS advantage for concurrent and sequential chemo-radiotherapy for p53abn disease compared to radiotherapy alone [30]. p53 is a poor prognosis biomarker, and further research is needed to identify precise markers to be used for prognostic and predictive purposes.

In the present study, we categorized EC patients into molecular subtypes by the NGS-based approach. Our EC cohort showed the following distribution of the four molecular subtypes: 5.9% POLE, 25.8% MMRd/MSI, 11.8% p53abn/TP53mut, and 56.5% NSMP. This distribution closely mirrored the proportions (6–12% POLEmut, 25–38% MMRd/MSI, 5–22% p53abn/TP53mut, and 28–60% NSMP) reported in other studies on unselected EC cases, which mainly had G1 and G2 endometrioid histology [6,31], demonstrating the discriminatory ability of our NGS model classifier. Furthermore, we correlated molecular subtypes to key clinical pathological features by univariate and multivariate analyses. Statistically significant differences between the groups were observed for histology, grade, FIGO stage, and LSVI. As previously reported [2,31,32], we found that the p53abn/TP53mut group was predominantly composed of III–IV-stage and high-grade serous histology, with substantial LVSI results (70%, Table 1, Figure 1). The second, most aggressive subtype was MMRd/MSI [32,33]. In our cohort, MMRd/MSI EC patients had primarily G2 (45%) and G3 (41%) endometrioid histology, often diagnosed at an early stage (86.4%). They also exhibited significantly lower LVSI values compared to the p53abn/TP53mut group, though the focal LVSI percentage (31.8%) was higher than that of the POLEmut and NSMP groups. In agreement with the previous studies [34], women belonging to the POLEmut group exhibited histopathologic features similar to those in the MMRd/MSI group, but without LSVI. Additionally, as previously reported [6,7,35,36], 5.8% of all EC cases fell into the multiple-classifier category harboring more than one molecular classifying feature and including POLEmut–MMRd/MSI, and MMRd/MSI–p53abn/TP53mut. The biological and prognostic significance of multiple-classifier ECs is unclear, and currently there is no consensus on how these tumors should be classified or treated. In our EC cohort, 56.5% of patients lacked the characteristics for molecular classification and were categorized into the NSMP group. This large and heterogeneous EC subtype presented a mix of clinical and histological characteristics, complicating the identification of clear prognostic or therapeutic patterns. Further in-depth studies are needed to refine and understand the molecular underpinnings of this EC group. Nevertheless, these results show that our NGS-based classification is a pragmatic and reliable approach for categorizing EC.

Regarding the MSI/MMR status assessment, in this study, multiple approaches were used to enhance the sensitivity and accuracy of the assay. Although MMR-IHC and MSI-PCR are often considered equivalent, discrepancies can arise due to differences in their detection mechanisms and in tumor histotypes [37,38,39]. When combined detection methods yield inconsistent results, NGS—proven to reliably distinguish MSI-H/MMRd from MSS/pMMR tumors—may be a valuable diagnostic tool. Previous research has already demonstrated the potential of NGS to identify MSI-H tumors and guide targeted immunotherapies [39]. In the current study, we compared IHC, Real-Time PCR, and NGS for detecting MSI/MMR status. Consistent with the previous findings [40,41], NGS and IHC demonstrated 97.6% concordance in identifying MSI-H/MMR status. Among the two cases of MSI subtype with discordant Real-Time PCR and NGS results, one case, with a loss of PMS2 staining but MSS by PCR, demonstrated consistency between NGS and IHC; the other case, with doubtful MSH6 staining by IHC and MSS by Real-Time PCR, revealed an MSI status through extensive NGS analysis. Indeed, it is known that in EC, MSH6 variations tend to result in a low MSI or MSS status, as these tumors exhibit a higher number of unstable events with mononucleotide repeats than dinucleotide repeats, which are more difficult to identify [42,43]. Another possible explanation for the conflicting results may be the inadequate proportion of tumor cells [44]. The concordance between NGS and IHC for p53 detection was 92.3%, consistent with the previous reports [15,16]. Only one case showed a discrepancy between genetic mutations and protein expression pattern, with *TP53* mutated and wild-type IHC staining. This finding suggests that the specific *TP53* mutation, typically resulting in a loss of function and often reduced or absent p53 protein expression, might have led to an aberrant p53 protein that was difficult to detect by IHC, or not consistently affect overall p53 levels. Taken together, these results highlight the feasibility and reliability of NGS analysis in distinguishing both MSI-H and *TP53* mutated tumors.

We further explored the potential of NGS as a tool to guide targeted treatment in EC. As the treatment landscape for EC rapidly evolves through the development of novel strategies [45,46,47,48], molecular characterization is becoming increasingly essential for determining potential therapeutic options. In our cohort, the 50-gene NGS panel revealed clinically significant variants, particularly in the *PIK3CA* (45 out of 85, 53%) and *KRAS* (18 of 85, 21%) genes (Figure 3). The relationship between *PIK3CA* mutations and prognosis in EC patients remains a topic of ongoing research, with mixed findings. Some studies indicated a favorable prognosis, others suggested an unfavorable prognosis [49,50,51]. The impact of *PIK3CA* mutations on prognosis may vary depending on factors, such as the specific domains of the gene affected, the mutation type, and the clinicopathological features of the individual patients. Moreover, it is essential to consider the broader genetic landscape of the tumor, as the co-presence of *PIK3CA* mutations with other alterations (e.g., *PTEN* mutations, MSI status, or *TP53* mutations) may also influence the overall prognosis. Given the complexity of and variability in outcomes, interpreting *PIK3CA* mutations in the context of comprehensive genetic testing and other clinical features is essential. In our EC cohort, NGS analysis revealed *PIK3CA* mutations that existed within different domains, some of which resulted in a gain-of-function protein that activated downstream signaling, while others were of unknown functional significance. These mutations have been observed across various cancer types, but they seem to present a distinct pattern in EC, strengthening the hypothesis that ECs exhibit a unique spectrum of somatic mutations in the *PIK3CA* gene, particularly in the amino-terminal domain of p110-α. When the effects of activating *PIK3CA* mutations on clinicopathological features were investigated, univariate analysis revealed a significant association with early FIGO stage (*p*-value 0.05, Table 2) and absent LSVI (*p*-value 0.01, Table 2), both of which are typically considered favorable prognostic factors in EC patients. In multivariate linear regression analysis, the association between *PIK3CA* mutations and LVSI remained significant, even after adjustments for other clinical variables, highlighting its potential prognostic impact (*p*-value = 0.05, Table 2). Additionally, *PIK3CA* mutations overlapped with *POLE* mutations. The co-occurrence with favorable clinicopathological features suggested that these alterations might represent a distinct molecular subset of EC associated with a better prognosis. Furthermore, we evaluated the distribution and relationship between *PIK3CA*, *PTEN*, and *KRAS* mutations (Figure 1F). The findings show overlapping occurrences of *PIK3CA* alterations with *PTEN* (*p*-value = 0.002) and with *KRAS* mutations (*p*-value = 0.93) in 78% and 39% of cases, respectively. While *PTEN* and *PIK3CA* mutations might play a synergistic role in EC, *KRAS* mutations can operate through distinct mechanisms. Some studies associate *KRAS* alterations with poor outcomes and aggressive behavior [51,52,53,54], but their impact on EC prognosis is still unclear. Further studies, including multivariate analyses and functional validation, are needed to confirm these associations and determine whether *PIK3CA*, *PTEN*, and *KRAS* mutations can serve as useful biomarkers for risk assessment in EC patients. The hypothesis that the PI3K pathway can be considered a target in EC treatment is becoming a reality. Several preclinical models are using PI3K signaling pathway inhibitors with variable results. The presence of *PIK3CA* and *PTEN* mutations in EC makes it unclear whether it increases sensitivity to PI3K signaling pathway inhibitors.

Interestingly, the identification of *ERBB2* (HER2) and *ESR1* variants in our cohort of NSMP patients may add valuable insights into potential therapeutic targets. The *ERBB2* amplification observed in three patients aligns with its established role in EC. HER2-positive tumors in EC can indeed respond well to anti-HER2 therapies, similar to breast cancer, providing a promising avenue for targeted treatment. Identifying such actionable variants in the NSMP subset, which can sometimes be challenging to classify molecularly, is exciting. Four ongoing clinical trials are evaluating the efficacy of HER2-targeted therapies in EC. The trials include serous and non-serous histology, advanced stages, p53 abn, and high grades of EC. Other studies tested the efficacy of HER2-targeted therapies in HER2-positive solid tumors (as an agnostic marker). The FDA recently approved fam-trastuzumab deruxtecan in HER2-positive (based on protein overexpression IHC 3+) solid tumors that had progressed [55]. With regard to HER2 protein expression analysis, in our study, only four patients had metastatic disease and underwent HER2 IHC testing. All four patients were HER2-negative by IHC, and these results are concordant with the absence of *ERBB2* amplification on NGS. Furthermore, *ERBB2*-amplified patients did not overlap with *TP53* mutations. This divergence from the previous findings [55,56] could reflect variations in histological types, limitations in sample size, or population-specific molecular characteristics. Regarding the *ESR1* gene (encoding for ERα), we observed hotspot mutations known to confer endocrine resistance. In primary EC patients, *ESR1* mutations in the ligand-binding domain have previously been associated with poorer prognosis and more aggressive disease [57,58]. These mutations were observed also in a setting of patients previously exposed to aromatase inhibitors, suggesting *ESR1* mutations may induce a mechanism of resistance to endocrine therapy in gynecologic malignancies [57,58]. These mutations are rare in early-stage breast cancer (<1%), but are acquired in up to 36% of cases that become resistant to aromatase inhibitors. The Utola DATA study shows that *ESR1* mutations are frequent in EC, suggesting the opportunity of hormonal treatment, especially in patients with recurrent/metastatic low-grade NSMP EC [59,60]. However, the impact of anti-ERα drugs on EC prognosis remains unclear. Future research should focus on developing improved ERα-targeting therapies or drug combinations that can better control EC while minimizing the side effects frequently associated with these treatments.

## 5. Conclusions

In conclusion, our study demonstrates the feasibility of applying a medium-complexity 50-gene NGS panel in clinical practice to support the molecular characterization of endometrial cancer. While the concordance between NGS and IHC was high, confirming the clinical applicability of the existing frameworks, such as the algorithm based on IHC plus *POLE* mutation testing, NGS technology emerges as a more complete and accurate approach. Our data also highlight additional molecular alterations—such as mutations in *PIK3CA*, *KRAS*, *ERBB2*, and *ESR1*—which may contribute to a deeper understanding of endometrial carcinogenesis. However, we acknowledge that these additional biomarkers, although potentially informative, are not currently clinically actionable and their relevance for guiding therapy remains exploratory. Further validation in larger, prospective, and multicenter cohorts is needed, ideally integrating multi-omics approaches and including more patients with advanced-stage or recurrent disease to assess therapeutic and prognostic implications more robustly. Despite these limitations, our findings provide a useful molecular portrait of endometrial cancer and a foundation for future studies aiming to expand the precision oncology landscape in this setting.

## Figures and Tables

**Table 1:** Clinicopathological features of EC patients and their associations to the molecular subtypes. Values are counts or mean ± standard deviation.

		Total Cases *n* = 85	POLEmut *n* = 5 (5.9%)	MMRd/MSI *n* = 22 (25.8%)	p53abn/TP53 *n* = 10 (11.8%)	NSMP *n* = 48 (56.5%)	Univariate Analysis *p*-Value	Multivariate Analysis *p*-Value
Age, years		64 ± 11	56 ± 10	63 ± 12	70 ± 12	64 ± 11	0.13	
Histotype								0.01
	Endometrioid	72 (84.7%)	5 (100%)	20 (91%)	3 (30%)	44 (92%)	<0.0001	
	Serous	8 (9.4%)	0	1 (4.54%)	6 (60%)	1 (2%)		
	Carcinosarcoma	4 (4.7%)	0	1 (4.54%)	1 (10%)	2 (4%)		
	Clear Cell	1 (1.2%)	0	0	0	1 (2%)		
Grade							<0.0001	0.01
	G1	10 (11.8%)	0	3 (13.5%)	0	7 (14.5%)		
	G2	48 (56.5%)	3 (60%)	10 (45.5%)	0	35 (73%)		
	G3	27 (31.7%)	2 (40%)	9 (41%)	10 (100%)	6 (12.5%)		
FIGO stage							0.007	0.03
	IA	26 (30.6%)	3 (60%)	4 (18.4%)	1 (10%)	18 (37.5%)		
	IB/II	36 (42.3%)	1 (20%)	15 (68%)	3 (30%)	17 (35.5%)		
	III	15 (17.7%)	1 (20%)	2 (9%)	2 (20%)	10 (20.8%)		
	IV	8 (9.4%)	0	1 (4.6%)	4 (40%)	3 (6.2%)		
Lymph node status							0.94	
	Negative	77 (90.6%)	5 (100%)	21 (95.5%)	9 (90%)	42 (87.5%)		
	Positive	8 (9.5%)	0	1 (4.6%)	1 (10%)	6 (12.5%)		
LVSI							0.02	0.05
	Absent	46 (54.1%)	3 (60%)	11 (50%)	2 (20%)	30 (62.6%)		
	Focal	18 (21.2%)	1 (20%)	7 (31.8%)	1 (10%)	9 (18.7%)		
	Substantial	21 (24.7%)	1 (20%)	4 (18.1%)	7 (70%)	9 (18.7%)		
Adjuvant Treatment						Survival		
None					50	Recurrence		0
Vaginal brachytherapy (VBRT)					2	Died of disease		0
External beam radiation therapy + vaginal brachytherapy (EBRT + VBRT)					19			
Chemo-radiotherapy + vaginal brachytherapy (CTRT + VBRT)					12			

**Table 2:** Univariable and multivariable association of *PIK3C* mutated subgroups with clinicopathological parameters. Values are counts or mean ± standard deviation.

		*PIK3CA* Activating Mutations (*n* = 31)	*PIK3CA* Unknown Mutations (*n* = 14)	*PIK3CA* Wild Type (*n* = 40)	Univariate Analysis *p*-Value	Multivariate Analysis *p*-Value
Age, years		61.8 ± 13	62.5 ± 11	67 ± 10	0.147	
Histotype					0.11	
	Endometrioid	30 (96.7%)	12 (85.7%)	30 (75%)		
	Serous	1 (3.2%)	2 (14.3%)	5 (12.5%)		
	Carcinosarcoma	0	0	4 (10%)		
	Clear cell	0	0	1 (2.5%)		
Grade					0.31	
	G1	4 (13%)	2 (14.3%)	4 (10%)		
	G2	18 (58%)	5 (35.7%)	27 (67.5%)		
	G3	9 (29%)	7 (50%)	9 (22.5%)		
FIGO stage					0.05	0.17
	IA	14 (45.2%)	3 (14.3%)	9 (25%)		
	IB/II	14 (45.2%)	4 (35.7%)	18 (45%)		
	III	3 (9.6%)	5 (35.7%)	7 (17.5%)		
	IV	0	2 (14.3%)	6 (12.5%)		
Lymph node status					0.36	
	Negative	30 (96.8%)	12 (85.7%)	35 (87.5%)		
	Positive	1 (3.2%)	2 (14.3%)	5 (12.5%)		
LVSI					0.01	0.05
	Absent	24 (77%)	4 (28.6%)	18 (45%)		
	Focal	4 (13%)	5 (35.7%)	9 (22.5%)		
	Substantial	3 (9.7%)	5 (35.7%)	13 (32.5%)		

## Data Availability

The data presented in this study are available upon request from the authors.

## Supplementary Materials

The following supporting information can be downloaded at: https://www.mdpi.com/article/10.3390/cancers17111806/s1, Table S1: NGS panel used in this study; Table S2: Sequencing metrics and alignment quality of the NGS runs; Table S3: Summary of PIK3CA mutations in ECs.
