# Supplementary material for: Clinical Application of Next-Generation Sequencing for Molecular Classification in the Management of Endometrial Cancer: An Observational Cohort Study
Source: Cancers (Basel). 2025 May 28;17(11):1806. doi: 10.3390/cancers17111806 (PMC12153688; doi:10.3390/cancers17111806)
Supplement: Supplementary file 1 [file cancers-17-01806-s001.zip › cancers-3605128-supplementary.pdf]

Table S1. NGS panel

Gene Name	SNVs/InDel	Exon	CNV	Transcript
AKT	X	4,5		NM_001014431.2
ALK	X	All (1-29)		NM_004304.5
AR	X	All (1-8)		NM_001011645.3
BRAF	X	11, 12, 15, 18		NM_004333.4
CDK4	X	2		NM_000075.2
CDKN2A	X	All (1-2)		More Transcripts
CTNNB1	X	3		NM_001904.4
DDR2	X	5, 8, 13, 14, 15, 16, 17, 18		NM_006182.4
EGFR	X	All (1-28)	X	NM_005228.5
ERBB2	X	All (1-27)	X	NM_004448.4
ERBB3	X	All (1-28)		NM_001982.4
ERBB4	X	10, 12		NM_005235.3
ESR1	X	5, 8		NM_001122740.2
FGFR1	X	All (2-18)		NM_001174067.2
FGFR2	X	All (2-18)		NM_000141.5
FGFR3	X	All (2-18)		NM_000142.5
FGFR4	X	3, 6, 9, 10, 12, 13, 15, 16		NM_213647.3
GNA11	X	4, 5		NM_002067.5
GNAQ	X	4, 5		NM_002072.5
GNAS	X	8		NM_016592
HRAS	X	2, 3, 4		NM_005343.4
IDH1	X	4		NM_005896.4
IDH2	X	4		NM_002168.4
KEAP1	X	All (2-6)		NM_203500.2
KIT	X	8, 9, 10, 11, 13, 14, 17, 18		NM_000222.3
KRAS	X	2, 3, 4		NM_004985.5
MAP2K1 (MEK1)	X	2, 3, 6		NM_002755.3
MET	X	2, 3, 4, 5, 6, 7, 8, 9, 10, 11, 12, 13, 14, 15, 16, 17, 18, 19, 20,21	X	NM_000245.4
MTOR	X	All (2-58)		NM_004958.4
NF1	X	All (1-58)		NM_001042492.3
NRAS	X	2, 3, 4		NM_002524.5
NTRK1	X	All (1-17)		NM_001007792.1
NTRK2	X	All (2-19)		NM_006180.5
NTRK3	X	All (3-20)		NM_001012338.3
PDGFRA	X	12, 14, 18		NM_006206.6
PIK3CA	X	2, 3, 5, 6, 8, 9, 10, 14, 21		NM_006218.4
POLE	X	1, 3, 4, 5, 6, 7, 8, 9, 10, 12, 13, 14, 19		NM_006231.4
PTEN	X	1, 2, 3, 4, 5, 6, 7, 8		NM_000314.8
RAF1	X	All (2-17)		NM_001354689.3
RB1	X	All (2-27)		NM_000321.3
RET	X	All (1-20)		NM_020975.6
ROS1	X	All (1-43)		NM_002944.3
SMAD4	X	All (2-12)		NM_005359.6
SMO	X	All (1-12)		NM_005631.5
STK11	X	All (1-9)		NM_000455.5
TERT	X	Promoter 1, 8, 9, 13		NM_198253.3
TP53	X	All (2-11)		NM_000546.6
TSC1	V	All (3-23)		NM_000368.5

MSI MARKERS BETHESDA		
**MSI sites**	**NM**	**hg19**
BAT25 (KIT)	NM_000222.2	chr4:55598212-55598236
BAT26 (MSH2)	NM_000251.2	chr2:47641560-47641586
CAT25 (CASP2)	NM_032983.4	chr7:143003343-143003367
MONO27 (MAP4K3)	NM_003618.4	chr2:39573063-39573089
NR21 (SLC7A8)	NM_012244.4	chr14:23652347-23652367
NR22 (STT3A)	NM_001278504.2	chr11:125490766-125490786
NR24 (ZNF3)	NM_001291604.2	chr2:95849362-95849384
NR27 (BIRC3)	NM_001165.5	chr11:102193509-102193534

OTHER MARKERS MSI		
**MSI sites**	**NM**	**hg19**
MSI_chr1_145002891_A_19		chr1:145002891-145002910
MSI_chr1_145260855_T_16		chr1:145260855-145260871
MSI_chr1_149900985_A_16		chr1:149900985-149901001
MSI_chr1_159290622_T_14		chr1:159290622-159290636
MSI_chr1_16572708_T_14		chr1:16572708-16572722
MSI_chr1_165793508_T_15		chr1:165793508-165793523
MSI_chr1_170733809_T_22		chr1:170733809-170733831
MSI_chr1_180075941_T_14		chr1:180075941-180075955
MSI_chr1_201754410_T_17		chr1:201754410-201754427
MSI_chr1_204084142_T_15		chr1:204084142-204084157
MSI_chr1_220318425_A_15		chr1:220318425-220318440
MSI_chr1_227618721_A_15		chr1:227618721-227618736
MSI_chr1_235486355_T_17		chr1:235486355-235486372
MSI_chr1_25685998_A_14		chr1:25685998-25686012
MSI_chr1_27621107_G_8		chr1:27621107-27621115
MSI_chr1_28618052_A_14		chr1:28618052-28618066
MSI_chr1_33402334_A_17		chr1:33402334-33402351
MSI_chr1_47325299_A_13		chr1:47325299-47325312
MSI_chr1_6257784_T_8		chr1:6257784-6257792
MSI_chr1_89449508_T_11		chr1:89449508-89449519
MSI_chr10_12381072_A_16		chr10:12381072-12381088
MSI_chr10_12424937_T_15		chr10:12424937-12424952
MSI_chr10_29760115_C_7		chr10:29760115-29760122
MSI_chr10_75630924_A_13		chr10:75630924-75630937
MSI_chr10_79635928_A_14		chr10:79635928-79635942
MSI_chr10_97918855_A_9		chr10:97918855-97918864
MSI_chr11_112832276_G_10		chr11:112832276-112832286
MSI_chr11_115047032_T_14		chr11:115047032-115047046
MSI_chr11_118220582_A_9		chr11:118220582-118220591
MSI_chr11_119545224_G_8		chr11:119545224-119545232
MSI_chr11_125763610_T_13		chr11:125763610-125763623
MSI_chr11_1490092_G_10		chr11:1490092-1490102
MSI_chr11_62649528_A_8		chr11:62649528-62649536
MSI_chr11_62703437_T_15		chr11:62703437-62703452
MSI_chr11_63149670_A_11		chr11:63149670-63149681
MSI_chr11_93170909_C_9		chr11:93170909-93170918

MSI_chr11_94151547_T_13		chr11:94151547-94151560
MSI_chr12_122242657_C_8		chr12:122242657-122242665
MSI_chr12_130785786_G_10		chr12:130785786-130785796
MSI_chr12_54405179_C_11		chr12:54405179-54405190
MSI_chr12_55759485_T_8		chr12:55759485-55759493
MSI_chr12_85285920_A_17		chr12:85285920-85285937
MSI_chr13_113965002_T_14		chr13:113965002-113965016
MSI_chr13_31722620_A_17		chr13:31722620-31722637
MSI_chr13_58299434_A_11		chr13:58299434-58299445
MSI_chr14_102821855_A_14		chr14:102821855-102821869
MSI_chr14_24040435_C_9		chr14:24040435-24040444
MSI_chr14_53513439_A_12		chr14:53513439-53513451
MSI_chr14_73959703_T_16		chr14:73959703-73959719
MSI_chr14_74054708_T_15		chr14:74054708-74054723
MSI_chr14_93708030_A_10		chr14:93708030-93708040
MSI_chr15_34157541_A_10		chr15:34157541-34157551
MSI_chr15_45897771_T_14		chr15:45897771-45897785
MSI_chr16_10867202_A_9		chr16:10867202-10867211
MSI_chr16_11051907_A_16		chr16:11051907-11051923
MSI_chr16_22650152_A_16		chr16:22650152-22650168
MSI_chr17_13981239_T_21		chr17:13981239-13981260
MSI_chr17_19314917_T_18		chr17:19314917-19314935
MSI_chr17_21322607_GT_34		chr17:21322607-21322641
MSI_chr17_27081792_T_16		chr17:27081792-27081808
MSI_chr17_52991731_A_16		chr17:52991731-52991747
MSI_chr17_56435160_C_7		chr17:56435160-56435167
MSI_chr17_74688717_A_15		chr17:74688717-74688732
MSI_chr18_511980_T_14		chr18:511980-511994
MSI_chr18_52445091_C_8		chr18:52445091-52445099
MSI_chr18_57013193_T_9		chr18:57013193-57013202
MSI_chr18_649879_T_15		chr18:649879-649894
MSI_chr19_14104688_T_14		chr19:14104688-14104702
MSI_chr19_49458970_G_8		chr19:49458970-49458978
MSI_chr19_49850472_G_8		chr19:49850472-49850480
MSI_chr2_148683685_A_8		chr2:148683685-148683693
MSI_chr2_165551295_A_9		chr2:165551295-165551304
MSI_chr2_203922057_A_9		chr2:203922057-203922066
MSI_chr2_207174427_A_9		chr2:207174427-207174436
MSI_chr2_242743804_C_6		chr2:242743804-242743810
MSI_chr2_39564893_T_28		chr2:39564893-39564921
MSI_chr2_48032740_T_13		chr2:48032740-48032753
MSI_chr2_62063093_A_17		chr2:62063093-62063110
MSI_chr2_97309468_T_16		chr2:97309468-97309484
MSI_chr20_46286320_A_10		chr20:46286320-46286330
MSI_chr20_47858503_A_8		chr20:47858503-47858511
MSI_chr20_61536691_A_11		chr20:61536691-61536702
MSI_chr22_26352178_T_16		chr22:26352178-26352194
MSI_chr22_29696468_T_16		chr22:29696468-29696484
MSI_chr22_37401594_T_15		chr22:37401594-37401609
MSI_chr3_100039735_A_9		chr3:100039735-100039744
MSI_chr3_113377481_T_11		chr3:113377481-113377492
MSI_chr3_130733046_T_11		chr3:130733046-130733057
MSI_chr3_157081226_T_9		chr3:157081226-157081235
MSI_chr3_30691871_A_10		chr3:30691871-30691881
MSI_chr3_37030780_T_11		chr3:37030780-37030791
MSI_chr3_51417603_C_7		chr3:51417603-51417610

MSI_chr3_71008341_T_13		chr3:71008341-71008354
MSI_chr4_39501722_A_18		chr4:39501722-39501740
MSI_chr4_83785564_T_9		chr4:83785564-83785573
MSI_chr5_122359502_AC_30		chr5:122359502-122359532
MSI_chr5_172421760_T_15		chr5:172421760-172421775
MSI_chr5_42495179_C_10		chr5:42495179-42495189
MSI_chr5_67584512_T_12		chr5:67584512-67584524
MSI_chr5_79970914_A_8		chr5:79970914-79970922
MSI_chr6_100382357_A_9		chr6:100382357-100382366
MSI_chr6_109954065_ATAG_40		chr6:109954065-109954105
MSI_chr6_11714639_A_14		chr6:11714639-11714653
MSI_chr6_142691950_T_17		chr6:142691950-142691967
MSI_chr6_43021976_G_12		chr6:43021976-43021988
MSI_chr7_17812488_C_8		chr7:17812488-17812496
MSI_chr7_77423459_T_9		chr7:77423459-77423468
MSI_chr8_134485098_A_14		chr8:134485098-134485112
MSI_chr8_17665797_T_15		chr8:17665797-17665812
MSI_chr8_23712066_T_12		chr8:23712066-23712078
MSI_chr8_37791833_T_9		chr8:37791833-37791842
MSI_chr8_79629738_A_14		chr8:79629738-79629752
MSI_chr9_121898657_G_11		chr9:121898657-121898668
MSI_chr9_136918528_G_8		chr9:136918528-136918536
MSI_chr9_33675364_A_9		chr9:33675364-33675373
MSI_chr9_95237024_TCA_44		chr9:95237024-95237068
MSI_chrX_101409254_T_16		chrX:101409254-101409270
MSI_chrX_37312610_C_8		chrX:37312610-37312618

Table S2. Sequencing metrics and alignment quality of the NGS runs

Runs	Reads (in millions)	Reads Passing Filter	%Q>30	Bases passing filter (in GB)
1	37,71	26,51	96,3	3,89
2	30,72	25,97	92,5	3.9
3	32,91	25,78	94,8	3,67
4	31,23	25,66	95,6	3,78
5	30,67	26,42	96,8	3,77
6	32,69	26,76	97,9	3,67
7	34,78	25,98	96,1	3,74
8	35,12	26,73	97,3	3,86
9	34,56	27,67	98,2	3,99
10	34,89	25,34	96,5	3,87
11	35,78	26,11	97,5	3,89

Sample Id	Clusters Mean Depth	Uniformity	Raw Reads	Valid Reads	DNA Valid Reads	% DNA Reads	% On Target	% Off Target	% Low Coverage Exonic Bases	Conformity
EC01	728	0,985	7127146	7125896	6020146	84,483	0,630	0,370	0,061	Compliant
EC02	1841	0,980	7642644	7642250	7171372	93,838	0,700	0,300	0,034	Compliant
EC03	693	0,993	4416912	4415270	4076176	92,320	0,610	0,390	0,050	Compliant

EC04	1184	0,990	6913020	6911958	6551886	94,791	0,620	0,380	0,035	Compliant
EC05	613	0,985	4503464	4502380	3237176	71,899	0,630	0,370	0,050	Compliant
EC06	1439	0,950	6197392	6196600	5237806	84,527	0,760	0,240	0,050	Compliant
EC07	1195	0,982	5463240	5459482	4253292	77,907	0,720	0,280	0,043	Compliant
EC08	1117	0,979	5181318	5166746	4701194	90,989	0,690	0,310	0,043	Compliant
EC09	1245	0,983	5374276	5320030	4137592	77,774	0,720	0,280	0,041	Compliant
EC10	627	0,987	4115866	4051718	3083842	76,112	0,630	0,370	0,068	Compliant
EC11	933	0,982	5131288	5054046	3796158	75,111	0,670	0,330	0,046	Compliant
EC12	1088	0,951	5673420	5591820	4040896	72,264	0,760	0,240	0,059	Compliant
EC13	1195	0,982	5463240	5439482	4253292	78,193	0,720	0,280	0,043	Compliant
EC14	943	0,989	7018486	6948222	4926684	70,906	0,660	0,340	0,044	Compliant
EC15	805	0,986	6152528	6033180	5072928	84,084	0,630	0,370	0,052	Compliant
EC16	635	0,985	6884374	6863104	5913266	86,160	0,610	0,390	0,059	Compliant
EC17	943	0,989	7018486	6948222	4626684	66,588	0,660	0,340	0,044	Compliant
EC18	805	0,986	6152528	6033180	4072928	67,509	0,630	0,370	0,052	Compliant
EC19	635	0,985	6884374	6765104	4913266	72,627	0,610	0,390	0,079	Compliant
EC20	943	0,989	7018486	6948222	4626684	66,588	0,660	0,340	0,044	Compliant
EC21	355	0,989	1418622	1418536	1275600	89,924	0,710	0,290	0,063	Compliant
EC22	415	0,983	1590974	1590732	1420408	89,293	0,720	0,280	0,038	Compliant
EC23	439	0,986	4422562	3422538	3375410	98,623	0,750	0,250	0,011	Compliant
EC24	711	0,972	2068318	2068210	1999102	96,659	0,770	0,230	0,056	Compliant
EC25	648	0,980	1872164	1872130	1807664	96,557	0,750	0,250	0,067	Compliant
EC26	466	0,985	1507686	1507604	1458746	96,759	0,730	0,270	0,011	Compliant
EC27	980	0,983	5136788	5034040	3889615	77,266	0,690	0,310	0,036	Compliant
EC28	1056	0,991	5466767	5429782	4253292	78,333	0,700	0,300	0,043	Compliant
EC29	1067	0,985	6876654	6745045	4013266	59,499	0,650	0,350	0,069	Compliant
EC30	986	0,975	5678901	5665431	4367581	77,092	0,690	0,31	0,065	Compliant
EC31	984	0,987	6878723	6676665	5677754	85,039	0,700	0,3	0,034	Compliant
EC32	897	0,988	4568763	4455575	4387488	98,472	0,690	0,31	0,023	Compliant
EC33	854	0,976	5675376	4563783	4127485	90,440	0,670	0,33	0,047	Compliant
EC34	875	0,986	7864563	7348458	4738394	64,481	0,630	0,37	0,046	Compliant
EC35	786	0,985	7658763	6847478	5676755	82,903	0,760	0,24	0,051	Compliant
EC36	965	0,980	6756435	5966098	4565432	76,523	0,720	0,28	0,048	Compliant
EC37	699	0,981	4577866	4476575	4187488	93,542	0,790	0,21	0,046	Compliant
EC38	769	0,979	4547473	4547231	4447654	97,810	0,720	0,28	0,039	Compliant
EC39	789	0,978	5467687	5447657	5347554	98,162	0,630	0,37	0,043	Compliant
EC40	879	0,986	5467098	5465778	5254678	96,138	0,670	0,33	0,054	Compliant
EC41	1135	0,991	7876655	7777555	7678274	98,723	0,760	0,24	0,042	Compliant
EC42	987	0,993	6879808	6865578	6836471	99,576	0,720	0,28	0,039	Compliant
EC43	934	0,992	6543230	6446757	6426375	99,684	0,660	0,34	0,034	Compliant
EC44	901	0,993	7656465	7654555	7625478	99,620	0,650	0,35	0,022	Compliant
EC45	879	0,987	5766444	5764574	5635485	97,761	0,680	0,32	0,059	Compliant
EC46	1237	0,985	5985438	5976545	5837485	97,673	0,660	0,34	0,044	Compliant
EC47	879	0,987	6549726	6548758	6462774	98,687	0,630	0,37	0,063	Compliant
EC48	1126	0,974	6789854	6776598	6527376	96,322	0,680	0,32	0,038	Compliant
EC49	1098	0,987	6237386	6236549	5633374	90,328	0,660	0,34	0,011	Compliant
EC50	1086	0,986	4565783	4564675	3964789	86,858	0,710	0,29	0,056	Compliant
EC51	996	0,985	6457354	6448765	5677832	88,045	0,720	0,28	0,067	Compliant
EC52	1236	0,984	4557685	4556765	3577865	78,518	0,750	0,25	0,059	Compliant
EC53	1145	0,987	5628375	5627658	4567859	81,168	0,770	0,23	0,023	Compliant
EC54	1135	0,984	6481293	6480654	5688886	87,783	0,750	0,25	0,033	Compliant
EC55	987	0,987	4517595	4507876	4456778	98,866	0,750	0,25	0,031	Compliant
EC56	976	0,988	7465753	7456546	6785544	91,001	0,690	0,31	0,021	Compliant
EC57	875	0,989	7345453	7334678	6858886	93,513	0,700	0,3	0,058	Compliant
EC58	995	0,986	7564324	7563465	6559279	86,723	0,650	0,35	0,036	Compliant
EC59	987	0,985	7461264	7460654	6339475	84,972	0,760	0,24	0,052	Compliant
EC60	945	0,984	6378457	6377688	5869242	92,028	0,750	0,25	0,043	Compliant
EC61	913	0,986	6472334	6467975	5478375	84,700	0,790	0,21	0,043	Compliant
EC62	934	0,978	5468583	5456789	4767378	87,366	0,720	0,28	0,052	Compliant
EC63	954	0,976	5876974	5867854	4778432	81,434	0,670	0,33	0,049	Compliant
EC64	965	0,984	5378327	5376875	4877543	90,713	0,690	0,31	0,044	Compliant
EC65	986	0,979	6347895	6346875	5634443	88,775	0,740	0,26	0,042	Compliant
EC66	1086	0,974	6435478	6434556	5688987	88,413	0,710	0,29	0,036	Compliant

EC67	1046	0,985	6238489	6229676	5996032	96,249	0,670	0,33	0,049	Compliant
EC68	989	0,978	6523825	6521356	5887792	90,285	0,660	0,34	0,043	Compliant
EC69	967	0,981	7567985	7558876	6582475	87,083	0,680	0,32	0,034	Compliant
EC70	912	0,982	7548934	7547765	7369487	97,638	0,680	0,32	0,047	Compliant
EC71	1062	0,986	6745966	6744559	6327495	93,816	0,640	0,36	0,039	Compliant
EC72	995	0,985	6253789	6252355	5938475	94,980	0,680	0,32	0,034	Compliant
EC73	934	0,987	7546563	7545786	6328428	83,867	0,670	0,33	0,032	Compliant
EC74	956	0,988	7845953	7834645	6789986	86,666	0,710	0,29	0,029	Compliant
EC75	867	0,986	7465654	7456998	6485829	86,976	0,790	0,21	0,044	Compliant
EC76	887	0,997	6783934	6776657	6273738	92,579	0,750	0,25	0,053	Compliant
EC77	846	0,987	6473856	6467778	5694858	88,050	0,780	0,22	0,038	Compliant
EC78	956	0,980	6839825	6838765	5737584	83,898	0,790	0,21	0,021	Compliant
EC79	923	0,985	6189385	6176659	5832847	94,434	0,740	0,26	0,034	Compliant
EC80	936	0,984	7237685	7224667	6237494	86,336	0,720	0,28	0,032	Compliant
EC81	987	0,983	5798696	5787767	5216364	90,127	0,650	0,35	0,040	Compliant
EC82	927	0,984	5343647	5342356	5127373	95,976	0,670	0,33	0,050	Compliant
EC83	976	0,987	5357587	5356658	5278521	98,541	0,720	0,28	0,043	Compliant
EC84	1089	0,987	6453539	6452543	6076474	94,172	0,790	0,21	0,044	Compliant
EC85	999	0,987	6453758	6452543	5737824	88,923	0,730	0,27	0,047	Compliant

Table S3. Summary of PIK3CA mutations in ECs.

Mutation	n =45	Effect on function	Type	Domain
**E81K**	1	Unknown	Missense	ABD
**R88Q**	5	Activating	Missense	ABD
**I102del**	1	Unknown	IF del	ABD
**K111E**	1	Activating	Missense	ABD
**E110del**	2	Unknown	IF del	ABD
**K111del**	2	Unknown	IF del	ABD
**V344A**	1	Unknown	Missense	C2
**C420R**	3	Activating	Missense	C2
**E545A**	1	Activating	Missense	Helical
**E545G**	3	Activating	Missense	Helical
**Y1021C**	6	Unknown	Missense	Kinase
**A1035V**	1	Unknown	Missense	Kinase
**M1043I**	4	Activating	Missense	Kinase
**H1047R**	12	Activating	Missense	Kinase
**H1047Y**	2	Activating	Missense	Kinase

ABD, adaptor-binding domain; C2, protein kinase-C homology 2; IF del, in-frame deletion.
